# Circular RNA screening from EIF3a in lung cancer

**DOI:** 10.1002/cam4.2338

**Published:** 2019-06-13

**Authors:** Ma‐Sha Huang, Fu‐Qiang Yuan, Yang Gao, Jun‐Yan Liu, Yi‐Xin Chen, Chen‐Jing Wang, Bai‐Mei He, Hong‐Hao Zhou, Zhao‐Qian Liu

**Affiliations:** ^1^ Department of Clinical Pharmacology Xiangya Hospital, Central South University Changsha P. R. China; ^2^ Institute of Clinical Pharmacology Central South University Hunan Key Laboratory of Pharmacogenetics Changsha P. R. China; ^3^ Xiangya Hospital, Central South University Changsha P. R. China

**Keywords:** bioinformatics, circular RNA, EIF3a, lung cancer

## Abstract

Eukaryotic initiation factor 3 (EIF3) is one of the largest and most complex translation initiation factors, which consists of 13 subunits named eukaryotic translation initiation factor 3 subunit A (EIF3a) to EIF3m. EIF3a is the largest subunit of EIF3. Previous studies suggested that EIF3a is a housekeeping gene, recent results have found that EIF3a is closely related to the tumorigenesis and drug resistance. Circular RNAs (circRNAs) derived from biologically important gene can play an important role in gene regulation. However, the mechanism underlying circRNAs’ biological functions is not well understood yet. In this work, we screened 31 EIF3a‐derived circRNAs, in which two circEIF3as were identified to be correlated with cisplatin drug sensitivity in lung cancer. Two circEIF3as were found involved in RNA‐binding proteins‐mediated biological processes and may be related to translational regulation according to bioinformatics analyses. CircEIF3as, the transcriptional initiation factor EIF3a transcribed circRNAs, are associated with both drug sensitivity and translation regulation. These findings mean that they may have a functional synergy effect with EIF3a or be valuable therapeutic targets for treatment like EIF3a. This is the first study that exploits circRNAs screening from EIF3a in lung cancer, our findings provide a novel perspective on the function of EIF3a and circEIF3as in lung cancer.

## 1 INTRODUCTION

Translation is the second step in the process of protein biosynthesis, and translation initiation is the first step in the translation process. The eukaryotic initiation factor 3 (EIF3) family plays an important role in eukaryotic translation. Moreover, eukaryotic translation initiation factor 3 subunit A (EIF3a) is the largest subunit of EIF3 family, which is a key player in all steps of translation initiation.^1^

Circular RNAs (circRNAs) are a class of RNAs that have a closed loop structure and distinct from traditional linear RNAs. Up to date, investigating circRNAs functions has become a global hot spot. Previous studies showed that circRNA could function as miRNA sponges and transcriptional regulators. In addition, they can be translated into proteins or interact with RNA‐binding proteins (RBPs).^2^

There have been reports that some genes and the circRNAs transcribed from them have biological functions at the same time. It is reported that DLG associated protein 4 (DLGAP4) is directly linked with brain diseases,^3^ such as early‐onset cerebellar ataxia,^4^ and circDLGAP4 may serve as a novel clinical treatment target for acute ischemic stroke.^5^ Increasing evidence indicates that homeodomain‐interacting protein kinase 3 (HIPK3) is a kinase gene in huntingtin disease,^6^, ^7^ and a recent study has found that silencing circHIPK3 significantly inhibits human cell growth.^8^ In addition, circHIPK3 can mediate the retinal vascular dysfunction in diabetes mellitus.^9^ Studies show that the circRNA transcribed from eukaryotic translation initiation factor 3 subunit J (EIF3J) can affect the expression and function of EIF3J.^10^ Poly (A) binding protein interacting protein 2 (PAIP2) can limit productive cytomegalovirus replication,^11^ and its expression is influenced by the circRNA transcribed from PAIP2.^10^

Considering the regulatory effects of circRNAs on their parental genes with biologically important function, these circRNAs are believed to have some biological functions. Moreover, circRNAs have both functional similarities and differences compared with their parental genes, and can even affect the function of their parental genes. In this case, we speculate that the circRNAs transcripts derived from EIF3a also play an important biological role in humans.

## 2 MATERIALS AND METHODS

### 2.1 Cell culture

The A549 human lung cancer cell line was purchased from the Chinese Academy of Sciences (Shanghai, China) and the A549/DDP human lung cancer drug‐resistant cell line was obtained from the cell biology research laboratory and Modern Analysis Testing Center of Central South University (Changsha, China). All the cell lines were maintained as adherent cell cultures in RPMI 1640 medium (Gibco, Life Technologies, USA) supplemented with 10% fetal bovine serum (FBS, 10099‐141; Gibco, MA, USA). Besides, the A549/DDP cell line was cultured in medium with 2 mg/L cisplatin (Sigma, P4394) to maintain the drug‐resistant phenotype before experimentation. Cells were cultured at 37°C in a humidified atmosphere of 5% CO_2_.

### 2.2 Drug sensitivity assay

Cells were seeded in 96‐well plates (4,000 cells/well) and cultured with different concentrations of cisplatin for 48 hours at 37°C in a humidified atmosphere of 5% CO_2_, followed by incubation with CCK‐8 solution. Then the cells’ viability was detected by measuring the absorbance at 450 nm using an Eon plate reader. The half‐maximal inhibitory concentration (IC_50_) was calculated from the relative survival curves.

### 2.3 RNA extraction, RNA RNase R treatment, and real‐time quantitative RT‐PCR

Total RNA was extracted using TRIzol (Invitrogen, CA, USA) according to the instructions. Cytoplasmic and nuclear RNAs were extracted using Cytoplasmic and Nuclear RNA Purification Kit (Norgen, Canada) according to the manufacturer's instructions. RNA RNase R treatment was carried out for 10 minutes at 37°C using 1ul RNase R (20 U/UL). Primescript RT Reagent Kit with gDNA Eraser (Takara Bio Inc, Japan) was used for synthesizing cDNA according to the manufacturer's instructions. CircRNAs expression was assessed by real‐time quantitative RT‐PCR using SYBR Premix Dimer Eraser assay kits. Real‐time quantitative RT‐PCR was performed through the Roche LightCycler 480 PCR System.

### 2.4 RNA immunoprecipitation assay

RNA immunoprecipitation assay (RIP) was performed using EZMagna RIP Kit (Millipore, Billerica, MA) according to the manufacturer's instructions. A549 cell lysates were incubated with anti‐rabbit AGO_2 _antibodies (Abcam, Cambridge, MA) or anti‐rabbit IgG antibodies (Millipore, Billerica, MA).

### 2.5 Western blot analysis

After transfection for 48 hours, western blot assay was carried out using the standard method, and then transferred onto polyvinylidene fluoride (PVDF) membranes (Millipore, Bedford, MA). After 2 hours of blocking, PVDF membranes were incubated with anti‐mouse β‐actin antibodies (Sigma‐Aldrich) and anti‐rabbit EIF3a antibodies (Cell Signaling Technology, USA) overnight at 4°C.

### 2.6 Differentially expressed gene analysis and survival analysis

We used THE HUMAN PROTEIN ATLAS (THPA) (https://www.proteinatlas.org/), THE CANCER GENOME ATLAS (TCGA) (https://cancergenome.nih.gov/) and ONCOMINE (https://www.oncomine.org) Database to identify the differential expression of EIF3a in various tissues and tumors. The prognostic value of EIF3a level in lung cancer and ovarian cancer are from KAPLAN‐MEIER PLOTTER (KMP) (http://kmplot.com/analysis/) Database.

### 2.7 Prediction for circEIF3as, circEIF3as‐RBPs axis and coding potential

CircEIF3as were predicted using CIRCBASE (http://circbase.org/) Database. The target RBPs of circEIF3as were predicted based on circRNA Interactome (https://circinteractome.nia.nih.gov/), RBPDB (http://rbpdb.ccbr.utoronto.ca/), catRAPID (http://service.tartaglialab.com/page/catrapid_group) and Cancer‐Specific CircRNA (http://gb.whu.edu.cn/CSCD/) Database. The graph of the circEIF3as‐RBPs axis was drawn with the Cytoscape 3_6_0. The Internal Ribosome Entry Site (IRES) and Open Reading Frame (ORF) of circEIF3as were predicted based on Circbank (http://www.circbank.cn/index.html) and ORF finder (https://www.ncbi.nlm.nih.gov/orffinder/) Database.

### 2.8 Functional enrichment analysis

During functional enrichment analysis of circEIF3as, the online analysis tool‐Metascape (http://metascape.org/gp/index.html#/main/step1) was utilized to perform gene ontology (GO) enrichment analysis.

### 2.9 Statistical analysis

The SPSS 19.0 software was used for general statistical analysis. Survival rate was calculated using the Kaplan‐Meier method.

### 2.10 Data availability statement

All data generated or analyzed during this study are included in this published article (and its Supplementary Information files).

## 3 RESULTS

### 3.1 The expression of EIF3a in tissues and tumors

**Figure 1:**
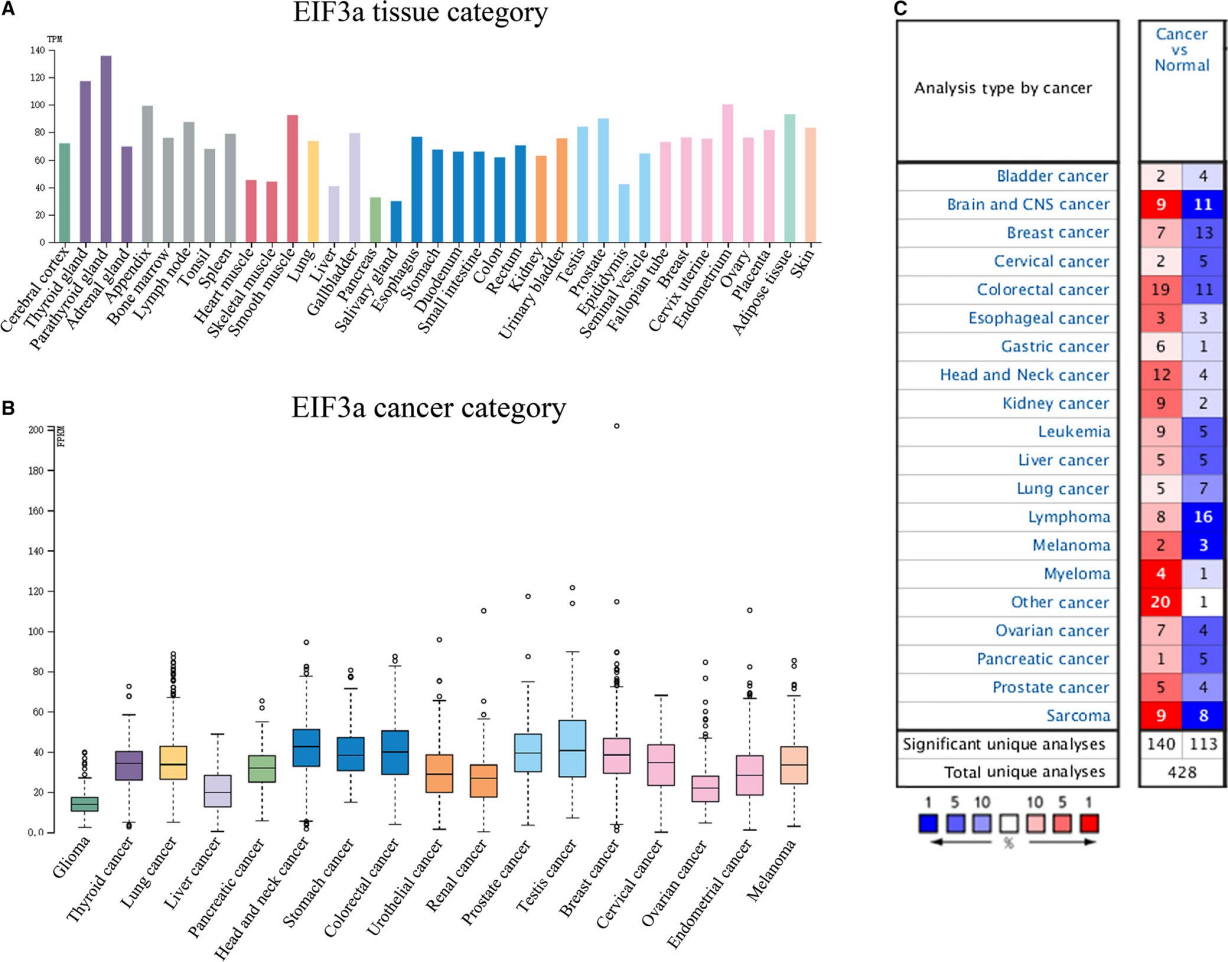
Panel A, EIF3a in different tissues, the data are obtained from THE HUMAN PROTEIN ATLAS (THPA) Database. Panel B, EIF3a in different cancers, the data are obtained from THE CANCER GENOME ATLAS (TCGA) Database. Panel C, The expression of EIF3a in various tumors, the data comes from ONCOMINE Database. EIF3a, eukaryotic translation initiation factor 3 subunit A

In previous studies, EIF3a was thought to be associated with the occurrence, metastasis, prognosis and treatment of cancer. It has been reported that EIF3a has biological function in lung cancer,^12^ breast cancer,^13^ pancreas cancer,^14^ gastric cancer,^15^ oral cavity cancer,^16^ colon cancer,^17^ esophagus cancer,^18^ ovary cancer,^19^ urinary bladder cancer,^20^ cervix cancer.^21^ Besides these, EIF3a is also emerging as a new potential anticancer drug therapeutic target in clinic, which improves the cisplatin sensitivity in lung cancer 22, 23, 24 and ovarian cancer.^19^ We use THPA Database to compare the expression level of EIF3a in different tissues (Figure 1A), where EIF3a tissue data are reported as a mean transcript per million experiment samples. The color‐coding is based on tissue groups, which consists of tissues with common functional features. The expression level of EIF3a in 17 cancer types is generated by the TCGA Database, in which the cancer types are color‐coded according to the type of normal organ that the cancer originates from (Figure 1B). The differential expression of EIF3a in various types of tumors is illustrated in the data from ONCOMINE Database (Figure 1C), where the red/blue color indicates high/low expression, respectively, and the darker color means higher/lower level of expression.

### 3.2 Association of prognostic value with EIF3a expression level

**Figure 2:**
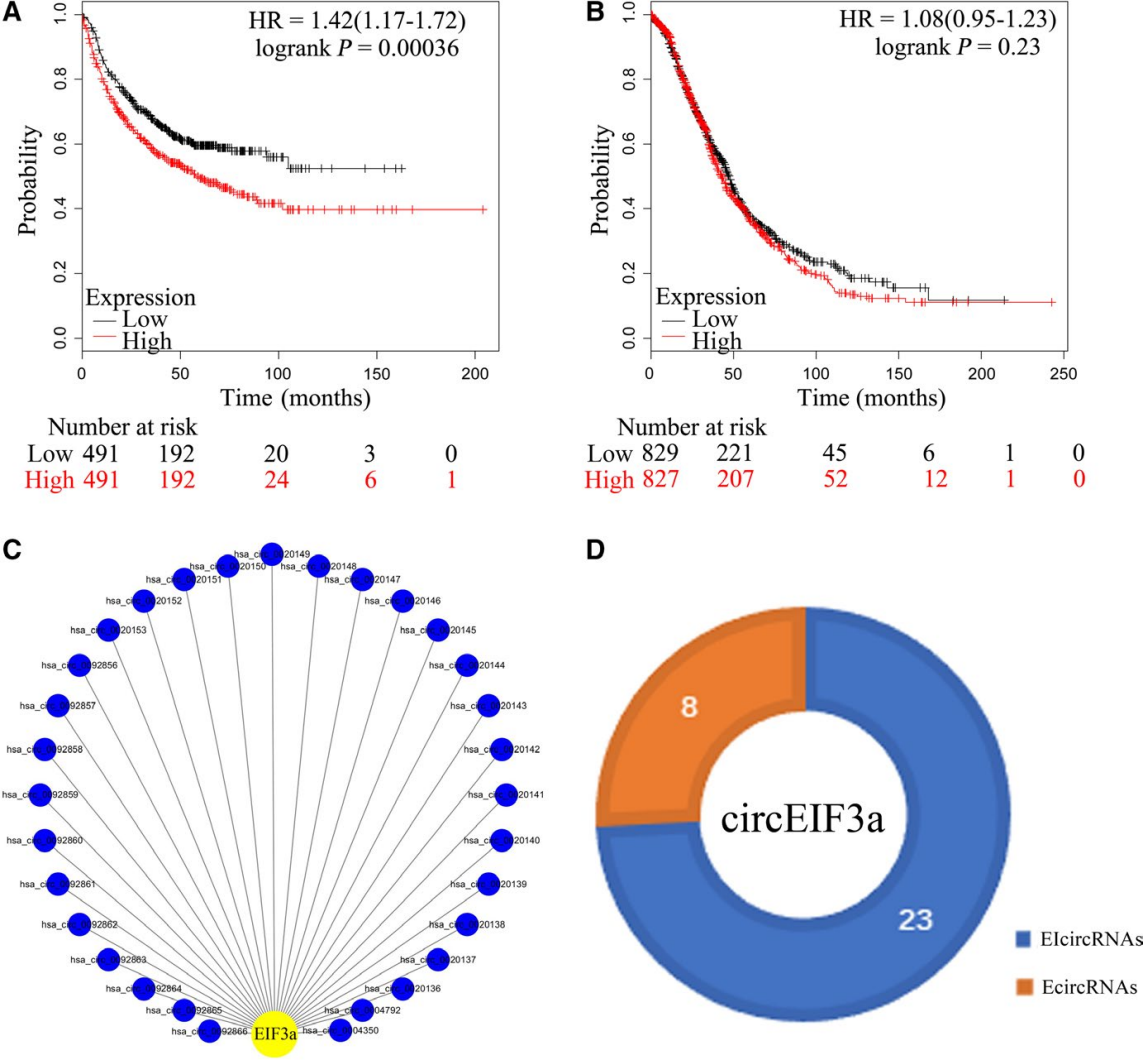
Panel A, The prognostic value of EIF3a level in lung cancer patients. Panel B, The prognostic value of EIF3a level in ovarian cancer patients. Panel C, Thirty‐one circRNAs derived from EIF3a. Panel D, The type and quantity of circEIF3as. EIF3a, eukaryotic translation initiation factor 3 subunit A. circRNA, circular RNA

As shown in Figure 1, EIF3a is highly expressed in various tissues and cancers, and on the other hand, EIF3a could present as both biomarkers and potential anticancer drug target in lung cancer and ovarian cancer.^19^, ^22^, ^23^, ^24^ Therefore, lung cancer and ovarian cancer will be taken as an example to analyze the impact of EIF3a on the survival of patients who suffered from these cancers. The results suggest that EIF3a has a significant effect on the survival rate of patients with lung cancer, but has little effect on the prognosis of patients with ovarian cancer (Figure 2A,B). The results are collected from KMP Database.

### 3.3 The circRNAs screening from EIF3a

Eukaryotic translation initiation factor 3 subunit A is a highly conserved gene located at 10q26.11 with 22 exons, spanning a 46‐kb DNA region.^25^ Human EIF3a is a 170‐kDa protein consisting of 1382 amino acids. According to the record in the CIRCBASE Database, a total of 31 circRNAs are derived from EIF3a, including 8 exon‐originated circular RNAs and 23 exons‐introns‐originated circular RNAs (Figure 2C,D, Table 1).

**Table 1:** Summary of circRNAs screening from EIF3a

	Circular RNA ID	Location	Genomic length	Spliced length	Type
1	hsa_circ_0004350	chr10:120832401‐120833449	1048	492	EIcircRNA
2	hsa_circ_0004792	chr10:120797749‐120797951	202	202	EcircRNA
3	hsa_circ_0020136	chr10:120794540‐120830597	36057	4618	EIcircRNA
4	hsa_circ_0020137	chr10:120796630‐120820356	23726	2692	EIcircRNA
5	hsa_circ_0020138	chr10:120797749‐120820356	22607	2501	EIcircRNA
6	hsa_circ_0020139	chr10:120809312‐120818909	9597	1215	EIcircRNA
7	hsa_circ_0020140	chr10:120810710‐120832565	21855	1942	EIcircRNA
8	hsa_circ_0020141	chr10:120810710‐120833449	22739	2270	EIcircRNA
9	hsa_circ_0020142	chr10:120818723‐120820840	2117	507	EIcircRNA
10	hsa_circ_0020143	chr10:120819113‐120819230	117	117	EcircRNA
11	hsa_circ_0020144	chr10:120819113‐120840334	21221	1589	EIcircRNA
12	hsa_circ_0020145	chr10:120820257‐120820356	99	99	EcircRNA
13	hsa_circ_0020146	chr10:120820257‐120820840	583	204	EIcircRNA
14	hsa_circ_0020147	chr10:120820257‐120833089	12832	1086	EIcircRNA
15	hsa_circ_0020148	chr10:120824910‐120825082	172	172	EcircRNA
16	hsa_circ_0020149	chr10:120824910‐120833089	8179	882	EIcircRNA
17	hsa_circ_0020150	chr10:120824910‐120833449	8539	1073	EIcircRNA
18	hsa_circ_0020151	chr10:120828957‐120830597	1640	409	EIcircRNA
19	hsa_circ_0020152	chr10:120832401‐120832565	164	164	EcircRNA
20	hsa_circ_0020153	chr10:120832401‐120833089	688	301	EIcircRNA
21	hsa_circ_0092856	chr10:120801769‐120802132	363	363	EcircRNA
22	hsa_circ_0092857	chr10:120809312‐120810833	1521	462	EIcircRNA
23	hsa_circ_0092858	chr10:120810710‐120816365	5655	237	EIcircRNA
24	hsa_circ_0092859	chr10:120810710‐120818909	8199	876	EIcircRNA
25	hsa_circ_0092860	chr10:120816251‐120816365	114	114	EcircRNA
26	hsa_circ_0092861	chr10:120816447‐120816552	105	105	EcircRNA
27	hsa_circ_0092862	chr10:120818723‐120830597	11874	1088	EIcircRNA
28	hsa_circ_0092863	chr10:120828957‐120833449	4492	901	EIcircRNA
29	hsa_circ_0092864	chr10:120830397‐120832565	2168	364	EIcircRNA
30	hsa_circ_0092865	chr10:120832542‐120833089	547	160	EIcircRNA
31	hsa_circ_0092866	chr10:120832952‐120833449	497	328	EIcircRNA

Abbreviation: EIF3a, eukaryotic translation initiation factor 3 subunit A.

### 3.4 Validation for the differential expression level and drug sensitivity of circEIF3as

**Figure 3:**
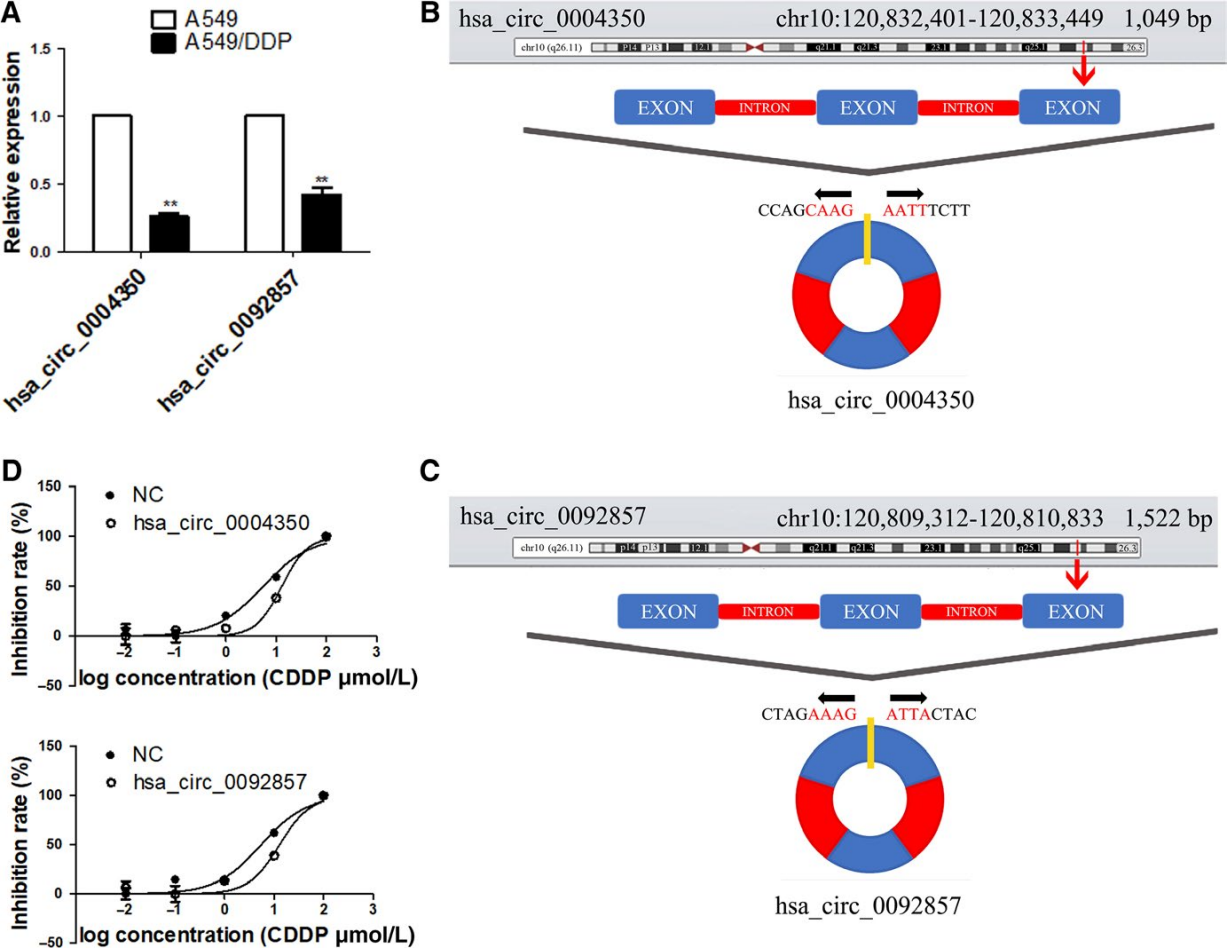
Panel A, The expression of hsa_circ_0004350 and hsa_circ_0092857 in A549 and A549/DDP cell line. (*P < 0.05; **P < 0.01). The diagram of position: hsa_circ_0004350 (panel B) and hsa_circ_0092857 (panel C). Panel D, The half‐maximal inhibitory concentration of hsa_circ_0004350 and hsa_circ_0092857

The circEIF3as is verified by real‐time quantitative reverse transcription‐PCR (qRT‐PCR) in A549 and A549/DDP cell line. We designed two pairs of primers to each circEIF3a for qRT‐PCR (Table S1). The results show that only two circRNAs transcribed from EIF3a (hsa_circ_0004350, hsa_circ_0092857) have differential expression in the two cell lines, and the rest are without differential expression or even with no expression in the two cell lines (Figure 3A). The locus of hsa_circ_0004350 is on chromosome 10:120.832.401‐120.833.449 in humans, while hsa_circ_0092857 is located on chromosome 10:120.809.312‐120.810.833 in humans. Both two circRNAs contain three exons and two introns (Figure 3B,C). The data were obtained from UNIVERSITY OF CALIFORNIA SANTA CRUZ GENOME BROWSER Database. Next, we explored whether hsa_circ_0004350 and hsa_circ_0092857 could affect cisplatin resistance in lung cancer cells, we found that down‐regulating the expression of hsa_circ_0004350 and hsa_circ_0092857 could affect cisplatin resistance in lung cancer cells (Figure 3D).

### 3.5 Possible biological functions of circEIF3as

**Figure 4:**
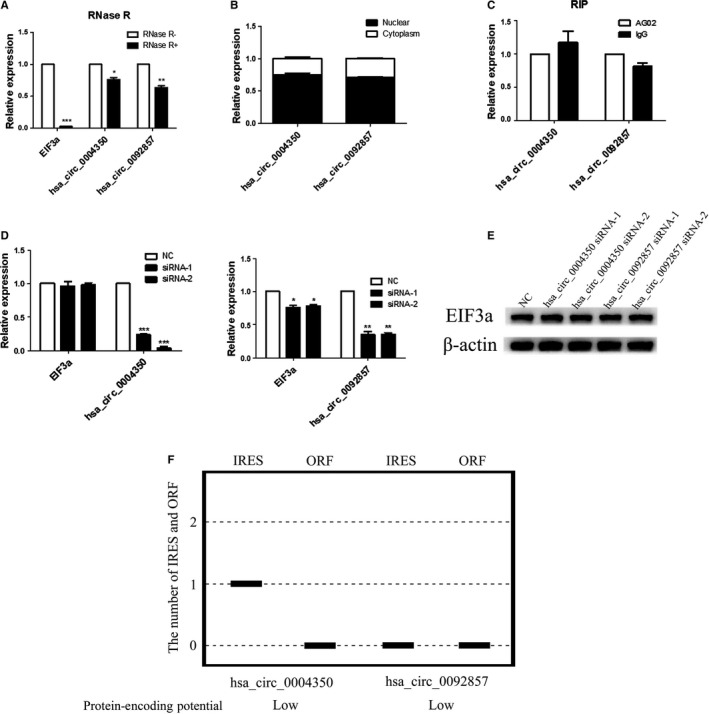
Panel A, After RNase R treatment, the expression levels of EIF3a and circEIF3as were determined by qRT‐PCR. Panel B, Subcellular fractionation assay was used to detect the localization of hsa_circ_0004350 and hsa_circ_0092857. Panel C, RIP assay indicating that hsa_circ_0004350 and hsa_circ_0092857 were not substantially accumulated in the AGO_2_ pellet. Hsa_circ_0004350 and hsa_circ_0092857 has little effect on the expression of EIF3a according to qRT‐PCR (panel D) and western blot (panel E) assays. Panel F, Protein‐encoding potential of circEIF3as. EIF3a, eukaryotic translation initiation factor 3 subunit A. EIF3a, eukaryotic translation initiation factor 3 subunit A; qRT‐PCR, quantitative reverse transcription‐PCR; RIP, RNA immunoprecipitation assay

Circular RNAs have different biological functions by acting as miRNA sponges or transcriptional regulators, and some of them are reported to have translation potential and can interact with RBPs. We conducted a series of experiments to determine the possible biological functions of circEIF3as. Compared with EIF3a mRNA, circEIF3as were resistant to RNase R digestion (Figure 4A). Subcellular fractionation assay indicated the cytoplasmic and nuclear enrichment and localization of circEIF3as (Figure 4B). RIP for AGO_2_ in A549 cells was performed, the results showed that circEIF3as were not accumulated in the AGO_2 _pellet (Figure 4C). In addition, we performed qRT‐PCR and western blot assays to investigate whether the circEIF3as can affect the expression of EIF3a (Figure 4D,E). In order to explore the translation potential of circEIF3as, we predicted the IRES and ORF via Circbank and ORF finder Database. Hsa_circ_0004350 has one IRES but no ORF, hsa_circ_0092857 has no IRES and ORF (Figure 4F). These data indicated that two potential circEIF3as were predominantly localized in the nucleus and were not accumulated in the AGO_2_ pellet. Furthermore, they had little effect on the expression of EIF3a and relatively low protein‐encoding potential. Taken together, we speculate that the two circEIF3as are involved in RBPs‐mediated biological processes.

### 3.6 Construction of the circEIF3as/RBPs axis regulation network

**Figure 5:**
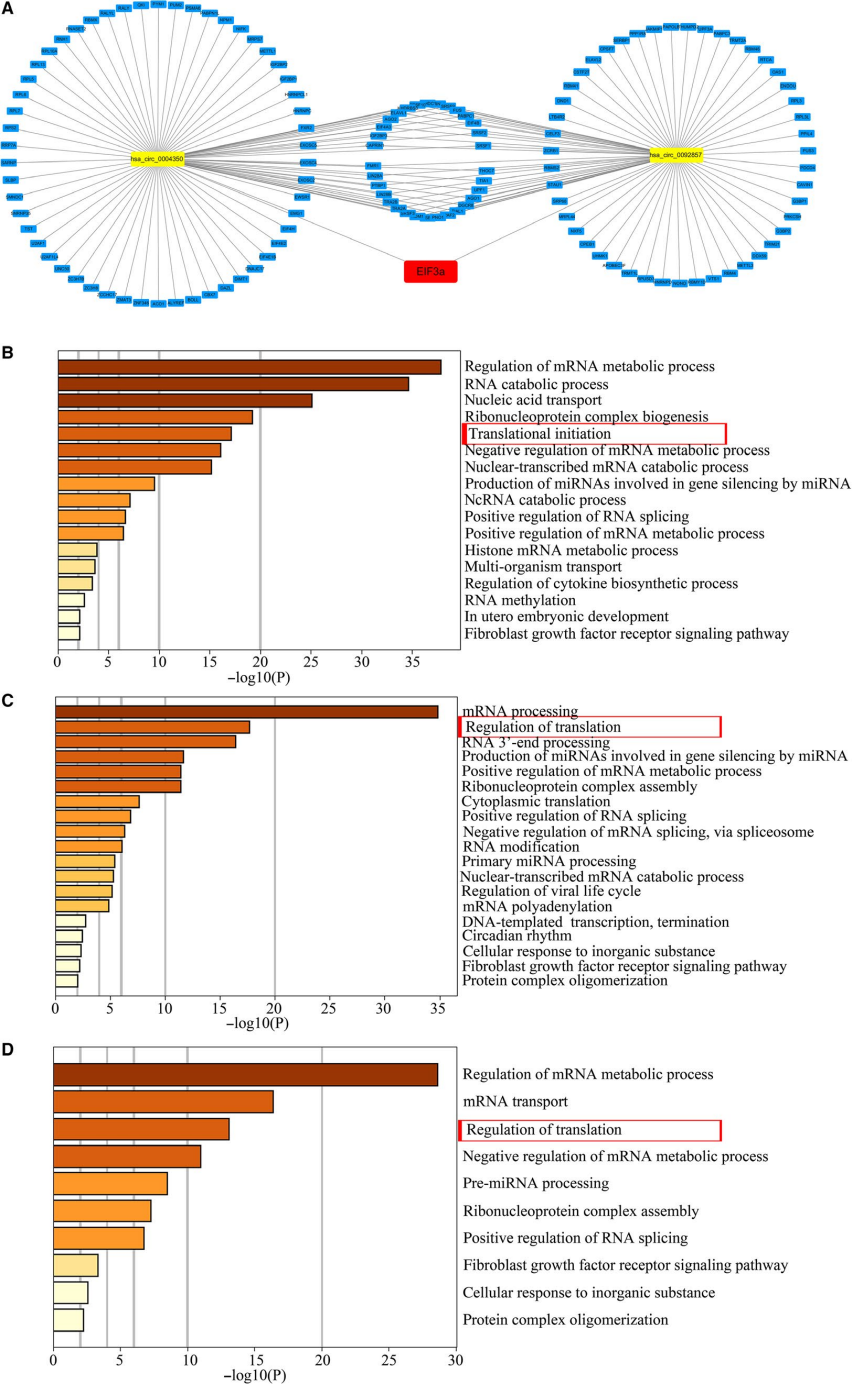
Panel A, The network of circEIF3as/RBPs axis. GO enrichment analysis of hsa_circ_0004350 (panel B) and hsa_circ_0092857 (panel C). Panel D, GO analysis on RBPs regulated by two circEIF3as. GO, gene ontology; RBPs, RNA‐binding proteins

To explore the function of two potential circEIF3as, we predicted the circEIF3as/RBPs axis via circRNA Interactome, RBPDB, catRAPID, and Cancer‐Specific CircRNA Database. We used the four databases to determine the target RBPs for each circEIF3a and combined the RBPs of four databases as the target for each circEIF3a (Table S2). The network of circEIF3as/RBPs axis is illustrated using Cytoscape (Figure 5A).

### 3.7 Functional enrichment analysis of circEIF3as/RBPs

To identify the biological roles of hsa_circ_0004350 and hsa_circ_0092857, we annotated their functions by the GO enrichment analysis. We used Metascape to predict the biological functions of hsa_circ_0004350 (Figure 5B) and hsa_circ_0092857 (Figure 5C). It is interesting to note that hsa_circ_0004350 and hsa_circ_0092857 are significantly associated with translation regulation. Next, we performed GO analysis on the overlapping RBPs regulated by two circEIF3as, we found that these overlapping RBPs were also regulators of translation (Figure 5D). These results suggest that the two circEIF3as may have a function synergy effect with the parental EIF3a gene.

## 4 DISCUSSION

In the past few years, with the development of high‐throughput sequencing and bioinformatics analysis, circRNAs have gradually become a research topic, which were once considered as transcriptional noise. CircRNAs have also been recognized as transcriptional regulators and miRNAs sponges. In addition, they can interact with RBPs and some of them can even be translated into proteins.

As the largest subunit of EIF3, EIF3a plays a bridging role in the initiation of translation. Numbers of studies have shown that EIF3a is involved in the initiation of mRNA translation in yeast cells and mammalian cells,^26^ and also regulates the initiation of protein translation. In the meantime, EIF3a is involved in the regulation of cell cycle,^27^ and shows a significant correlation with cell growth 28 and differentiation.^29^ Recent studies have found that EIF3a is closely related to the occurrence, development, prognosis and chemotherapy efficacy of tumors.^17^ Moreover, EIF3a gene polymorphism is also associated with the susceptibility of malignant tumors.^30^

The above results indicate that EIF3a exerts a crucial influence on the physical fitness of human beings. EIF3a can be transcribed into both mRNA and circRNAs. Until now, there is no report about circRNAs transcribed from EIF3a. Therefore, we bravely assumed that circEIF3as has the biological function. We detected the expression of circEIF3as in the lung cancer cell line and lung cancer drug‐resistant cell line of human, the results showed that hsa_circ_0004350 and hsa_circ_0092857 had differential expression in two cell lines. Moreover, we found that down‐regulating the expression of hsa_circ_0004350 and hsa_circ_0092857 could affect cisplatin resistance in lung cancer cells. By using bioinformatical methods, we predicted the RBPs of these two circEIF3as in four databases. Next, we performed GO enrichment analysis of hsa_circ_0004350 and hsa_circ_0092857 to annotate the biological functions, we found that the two circEIF3as were related to translation regulation, showing that they may have a function synergy effect with EIF3a.

In conclusion, we systematically analyzed the expression of EIF3a in various tissues and tumors, and also discussed the prognostic value of EIF3a in lung cancer patients and ovarian cancer patients. Our study for the first time revealed the expression signatures of the circRNAs transcripts derived from EIF3a in lung cancer. Furthermore, we established the biological functions of circEIF3as with different bioinformatical analyses. The further studies on functions and mechanisms underlying hsa_circ_0004350 and hsa_circ_0092857 are being carried out in our laboratory. Our findings support that hsa_circ_0004350 and hsa_circ_0092857 are derived from the translation initiation factor‐EIF3a, moreover, they are involved in drug sensitivity and translational regulation. These results suggest that these two circEIF3as may have functional synergy with their parental gene‐EIF3a, and may also serve as potential targets for lung cancer treatment like EIF3a. These data provide the basis for further studies on the diagnosis, treatment and biological function of circEIF3as in lung cancer.

## CONFLICT OF INTEREST

The authors declare no conflict of interest.
